# Autoimmune Hepatitis Following mRNA COVID-19 Vaccination in a Very Old Patient With Preexisting Sjögren’s Syndrome: A Case Report

**DOI:** 10.7759/cureus.30896

**Published:** 2022-10-31

**Authors:** Yukinari Yoshida, Norikazu Iwata, Yoshifumi Ishii, Yuji Hinoda, Takao Endo

**Affiliations:** 1 Department of Internal Medicine and Gastroenterology, Sapporo Shirakabadai Hospital, Sapporo, JPN; 2 Department of Pathology, Sapporo Shirakabadai Hospital, Sapporo, JPN

**Keywords:** very old patient, sjögren’s syndrome, mrna covid-19 vaccine, sars-cov-2, autoimmune hepatitis

## Abstract

A case of autoimmune hepatitis (AIH) following COVID-19 vaccination in a very old patient is presented. An 85-year-old woman who had preexisting Sjögren’s syndrome (SS) but had never shown evidence of liver disease was admitted to our hospital due to jaundice and liver dysfunction. Further laboratory tests, imaging studies, and liver histology proved this to be a case of definite AIH. Eight weeks before the disease onset, she had received the second dose of mRNA COVID-19 vaccination. To our knowledge, this is the first case of AIH following COVID-19 vaccination in a patient with a history of SS.

## Introduction

Amid the ongoing pandemic of SARS-CoV-2 infection, causing COVID-19, which first occurred in December 2019, new variants of the virus are appearing one after another. In an attempt to control the pandemic, a mass vaccination campaign has been vigorously conducted worldwide, including in Japan, and booster doses are being recommended. More recently, autoimmune diseases, including autoimmune hepatitis (AIH) following COVID-19 vaccination have been increasingly reported, which developed in both people with or without an autoimmune history [1-5]. Despite their rarity, these reports raise awareness of the potential causality between the two and the possible underlying mechanism, but we still lack an adequate understanding of them. To resolve this issue, further accumulation of similar cases and analysis of clinical and epidemiological data continuously are necessary, although experimental investigation can be expected.

AIH constitutes a representative liver disease associated with autoimmunity, and Sjögren’s syndrome (SS) is a systemic rheumatic disease characterized by dryness of the eyes and mouth, resulting from autoimmune-induced inflammation of the lacrimal and salivary glands. Of the reported cases of AIH after COVID-19 vaccination, about 30% had an autoimmune history [4,5]. The first reported case of AIH following mRNA COVID-19 vaccination in a patient with preexisting SS is presented. The patient is also the oldest of the previously reported cases of AIH after COVID-19 vaccination and is, thus, a very important case.

## Case presentation

**Figure 1:**
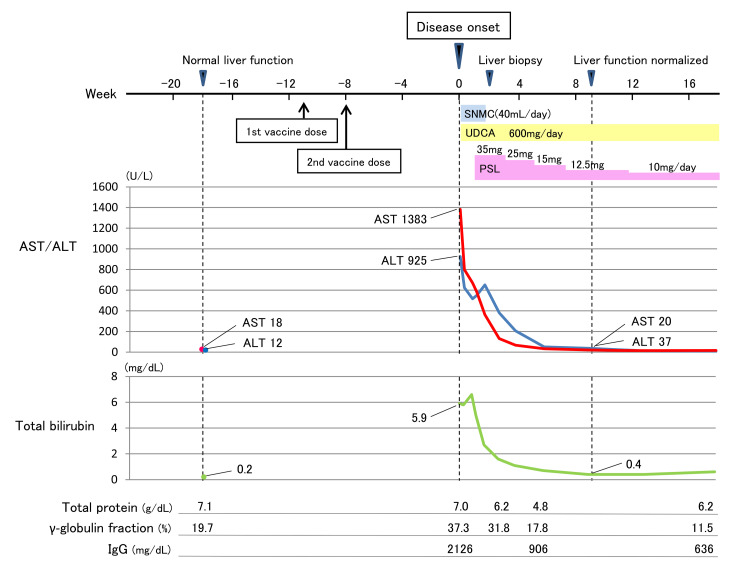
Clinical time course and evolution of laboratory findings. Intravenous injections of SNMC (40 mL/day) and oral UDCA (600 mg/day) were started at the disease onset. The former ended two weeks later, and the latter continued. Oral PSL (35 mg/day) was started one week after the onset, and the dose was subsequently tapered. AST, aspartate aminotransferase; ALT, alanine aminotransferase; IgG, immunoglobulin G; SNMC, Stronger Neo-Minophagen C; PSL, prednisolone; UDCA, ursodeoxycholic acid

In the fall of 2021, an 85-year-old Japanese woman visited our hospital with complaints of jaundice. She was our regular outpatient with hypertension for two years, and in addition, her history included SS diagnosed according to the Revised Japanese Criteria for SS at another hospital and lacunar strokes at 77 and 80 years of age, respectively. However, she did not have a history of liver disease. She had been prescribed amlodipine 5 mg daily for hypertension and aspirin 100 mg daily for the prevention of cerebral infarction. Her SS involved mild sicca syndrome, for which she was receiving no treatment. Her clinical time course and laboratory findings are summarized in Figure 1.

Blood tests showed aspartate aminotransferase 1383 U/L, alanine aminotransferase 925 U/L, alkaline phosphatase 207 U/L, gamma (γ)-glutamyltransferase 326 U/L, and total bilirubin 5.9 mg/dL. Her white blood cell count was normal without eosinophilia, and her serum C-reactive protein was slightly positive (1.07 mg/dL), but the γ-globulin fraction was markedly elevated (37.3%). On previous routine checkups, including the latest checkup four months (18 weeks) earlier, the absence of liver dysfunction and hyper-γ-globulinemia was confirmed. After the last tests, as before, she maintained a very strictly limited lifestyle to prevent exposure to communicable diseases, including COVID-19, and she continued her days, as usual, starting no new medications or supplements, other than vaccination against COVID-19. The patient had received two doses of the mRNA COVID-19 vaccine, BNT162b2 (Pfizer/BioNTech, New York, NY, USA), encoding the whole spike protein of SARS-CoV-2, the first dose 11 weeks and the second dose 8 weeks before the disease onset. She did not drink alcohol. The nasal swab polymerase chain reaction test for SARS-CoV-2 was negative. The patient was thus hospitalized for further evaluation.

**Figure 2:**
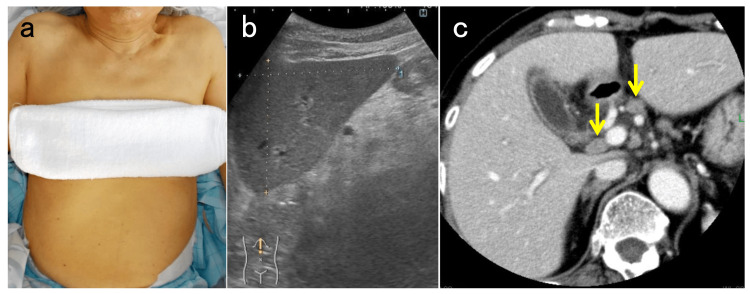
Skin appearance of the patient and findings of imaging studies of the abdomen. (a) Icteric skin is observed. (b) Ultrasound shows dullness of the edge of the left hepatic lobe, indicative of mild liver swelling. (c) On contrast-enhanced computed tomography, enlarged reactive lymph nodes (yellow arrows) are seen at the hepatic hilus.

On physical examination, she showed only icterus, with no fever and no abnormal findings of the chest and the abdomen. On both ultrasound and contrast-enhanced computed tomography of the abdomen, mild hepatomegaly and enlarged reactive lymph nodes at the hepatic hilus were seen (Figure 2).

**Figure 3:**
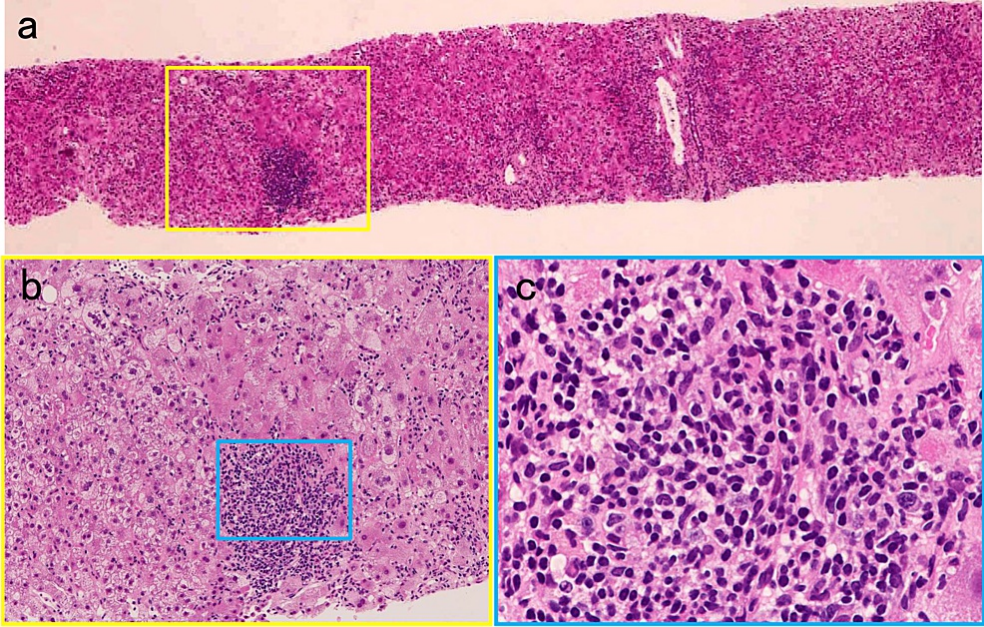
Histopathological findings of the liver biopsy (hematoxylin-eosin staining). (a) Low magnification showing mainly portal tract inflammation and mild lobulitis, partly with the ballooning change of hepatocytes (40×). (b) Magnified image of the yellow square in (a) showing high-grade inflammation in the portal area, with the destruction of the limiting plate and invasion to the lobular parenchyma by infiltrated mononuclear cells, indicating interface hepatitis (200×). (c) A high-magnification image of the blue square in (b) showing intense lymphocyte infiltration with plasma cells (400×).

The serological profile of hepatitis-related viruses was negative for hepatitis A, B, C, and E viruses, cytomegalovirus, and Epstein-Barr virus. Total IgG was increased to 2126 mg/dL (normal range 820-1740 mg/dL). Because the patient had SS as a preexisting autoimmune condition, with positive antinuclear antibody (1:1280, speckled pattern), rheumatoid factor (64 U/mL), and anti-SSA/Ro antibody (1:64), which were once checked at our hospital in the summer of 2020, the liver dysfunction with hyper-γ-globulinemia was presumed to be associated with autoimmunity. Regarding other autoantibodies, only the anti-smooth muscle antibody was found to be marginally positive (1:20). Liver biopsy was eventually performed after stopping aspirin. On liver histology, high-grade infiltration of lymphocytes with an admixture of plasma cells in the portal area extending into the lobular parenchyma, indicating interface hepatitis, was seen (Figure 3).

Slight periportal fibrosis was observed, suggestive of the acute hepatitis phase. According to the Revised International AIH Group criteria, the patient’s pretreatment score was 20 (>15 suggests definite AIH); therefore, the diagnosis was definite AIH. Treatment with oral prednisolone (PSL) 35 mg/day was started one week after onset because the total bilirubin tended to increase, and the diagnosis of AIH had already been made with a revised score for AIH of 16 before liver biopsy. Due to the good response, the dose of PSL was subsequently tapered. Her liver enzymes and total bilirubin normalized eight weeks later, and total IgG was also confirmed to be within the normal range four weeks later. Ten months after the start of treatment, the patient’s liver function remained normal with a maintenance PSL of 4 mg/day.

## Discussion

Since the beginning of the pandemic caused by SARS-CoV-2, which occasionally produces immune dysregulation in the host, cases of diverse immune-mediated diseases during/after COVID-19 have been reported [6]. Similarly, reported cases of autoimmune diseases following COVID-19 vaccination, including AIH, have recently increased [2,3]. In April 2021, the first case report of AIH by Bril et al. after COVID-19 vaccination was published online [1], and so far, there have been more than 30 similar cases in the English medical literature [4,5].

It is commonly postulated that autoimmune disease develops in genetically predisposed individuals following exposure to environmental triggers, leading to the breakdown of immune tolerance to autoreactive lymphocytes. The patient in the present case had preexisting SS, indicating her susceptibility to an autoimmune response. It is well known that AIH accompanies other autoimmune diseases, such as Hashimoto’s thyroiditis (8%), SS (7%), and rheumatoid arthritis (3%) [7]. Of the reported cases of AIH after COVID-19 vaccination, three were found to have Hashimoto’s disease [8-10], whereas to our knowledge, this is the first case with a history of SS. In addition, mRNA COVID-19 vaccination was possibly the trigger for the AIH in the present case, as follows. This very old patient had shown normal liver function at least four months before onset, and the only unusual event in her daily life during the four months before disease onset was COVID-19 mRNA vaccination. Except for the vaccination, no environmental factors linked with AIH onset could be identified. Together with her predisposing factor described above, these suggest a causal relationship between the COVID-19 vaccination and AIH, although a merely coincidental co-occurrence cannot be denied.

A plausible mechanism by which viral infection induces autoimmunity is molecular mimicry between viral and self-antigens. Indeed, in 2020, Vojdani and Kharrazian reported an evaluation of antigenic cross-reactivity between SARS-CoV-2 proteins, including the spike protein and human tissue antigens [11]. Nonspecific bystander activation of dormant autoreactive T cells may also be involved in the autoimmune response. Autoimmunity induced by mRNA COVID-19 vaccination can arise based on similar mechanisms through the produced spike protein, whereas the mRNA of the vaccine itself is predicted to stimulate innate immunity via Toll-like receptor (TLR) 7, which is expressed by B cells, along with dendritic cells, and recognize single-stranded RNAs, thereby triggering activation of autoreactive B cells in a T-cell-independent manner [12,13]. Interestingly, it has most recently been hypothesized that age-associated B cells (ABCs), which belong to the memory compartment, a unique subset of B cells expressing CD11c and T-bet, are potentially induced by mRNA COVID-19 vaccines and may be implicated in autoimmune phenomena [14]. ABCs are increased during aging, infection, and autoimmunity, such as several autoimmune diseases, including SS [15,16]. Of note, ABCs are shown to be hyper-responsive to TLR7 signaling and are poised to generate autoreactive antibody-secreting plasmablasts in systemic lupus erythematosus [17].

In this case, after COVID-19 vaccination, serum γ-globulin was markedly elevated, a hallmark of AIH, indicating the excessive stimulation of plasma cells. Setting aside the question of whether the plasma cell differentiation depends on T cells, this suggests that the B-cell-driven immune response may have been closely involved in the pathogenesis of AIH in this case, wherein autoreactive B cells to hepatocytes could be activated by TLR7 stimulation via mRNA vaccination to produce specific autoantibodies that may participate in the immune-mediated liver injury. This proposed mechanism could be supported by the evidence that ABCs expanded in patients with SS not only produce antibodies specific to various self-antigens but are also responsive to TLR stimulation [15,16].

The time to AIH onset from COVID-19 vaccination varies across the reported cases, ranging from two days to two months (mean time 21.1 days) after the first vaccination [5]. This could be derived from the heterogeneity of AIH itself, in addition to the different vaccine agents administered. The latency time of the present case, 8 weeks after the second dose (or 11 weeks after the first dose) of the vaccination, seemed to be long compared with the other cases reviewed in 2022 [4,5]. It is possible that there could be a delay in the recognition of onset as the present patient was asymptomatic other than the sign of jaundice, or immune-mediated hepatitis might have shown relatively slow progression.

As with the majority of reported cases of AIH following COVID-19 vaccination, the present case responded well to corticosteroid therapy, with dramatic improvements in hyper-γ-globulinemia and liver dysfunction. However, clinicians need to be aware that three cases (about 10%) of liver failure have been documented: one of them who received BNT162b2 was alive owing to liver transplantation [18], and the other two who received ChAdOx1 nCoV-19, an adenovirus vector vaccine, died [19,20].

Of the 31 reported cases previously summarized by Lleo et al. [4], ranging from 32 to 80 years of age (median 58 years), 3 were 80 years old, and so far, the present patient is the oldest. The vast majority of very old people (e.g., aged over 85 years) in many nations have been administered mRNA-based or adenovirus-based vaccines in the real-world setting; therefore, the adverse effects of these types of vaccines, including autoimmune phenomena, in these people are yet to be reported in detail.

## Conclusions

In summary, a possible case of AIH -triggered by COVID-19 mRNA vaccination in a very old patient with preexisting SS, who had a striking recovery with corticosteroid therapy, was presented. AIH after COVID-19 vaccination appears to be exceedingly rare, and the causality between the two cannot be definitively established at present. Thus, the overwhelming benefits of mass COVID-19 vaccination are not undermined by the autoimmune event. Nonetheless, the incidence of AIH following COVID-19 vaccination and the causal link will be further elucidated by global pharmacovigilance, especially those in patients with preexisting autoimmune diseases and the very old population need to be clarified.
